# Effect of Soil Moisture Content on the Splash Phenomenon Reproducibility

**DOI:** 10.1371/journal.pone.0119269

**Published:** 2015-03-18

**Authors:** Magdalena Ryżak, Andrzej Bieganowski, Cezary Polakowski

**Affiliations:** Institute of Agrophysics, Polish Academy of Sciences, Lublin, Poland; NERC Centre for Ecology & Hydrology, UNITED KINGDOM

## Abstract

One of the methods for testing splash (the first phase of water erosion) may be an analysis of photos taken using so-called high-speed cameras. The aim of this study was to determine the reproducibility of measurements using a single drop splash of simulated precipitation. The height from which the drops fell resulted in a splash of 1.5 m. Tests were carried out using two types of soil: Eutric Cambisol (loamy silt) and Orthic Luvisol (sandy loam); three initial pressure heads were applied equal to 16 kPa, 3.1 kPa, and 0.1 kPa. Images for one, five, and 10 drops were recorded at a rate of 2000 frames per second. It was found that (i) the dispersion of soil caused by the striking of the 1st drop was significantly different from the splash impact caused by subsequent drops; (ii) with every drop, the splash phenomenon proceeded more reproducibly, that is, the number of particles of soil and/or water that splashed were increasingly close to each other; (iii) the number of particles that were detached during the splash were strongly correlated with its surface area; and (iv) the higher the water film was on the surface the smaller the width of the crown was.

## Introduction

Soil, being the top layer of the Earth's crust and a component of many ecosystems, undergoes continuous degradation [1,2]. One of the forms of this degradation is water erosion [3–5], the first step of which is the splash phenomenon. This is caused by the impact of a water drop on the surface of soil. This impact results in disintegration of soil aggregates, generation of sediment to transport, acceleration of surface seal formation, decreased infiltration, and increased overland flow [6]. Splash can be characterized by two sub-processes: the detachment of the particles from the surface and the transportation of these particles in random directions [7]. The natural places in which research of the splash phenomenon can be conducted are field conditions using natural rainfall. In field conditions the following parameters were measured: the amount of splashed material using splashcups [8], height of splash during the rain [3], and travel distances of particles experiencing rainsplash [9]. The methodologies used to study rain splash and wash processes were also elaborated [4]. In order to compare the dynamics of interrill soil erosion processes a rainfall simulator in both laboratory and field scales was used [10]. The nature of the impact of water drops with a soil surface is complex and depends on many factors, such as: drop size, impact velocity, soil texture, grain shape, and packing density [11]. Hence, splash measurements are more likely conducted in the laboratory, where it is easier to control these parameters and reduce the number of variables. Using a rainfall simulator, Leguédois et al. [12] measured the breakdown of soil aggregates under the influence of precipitation and their transportation to a specific distance. Jomaa et al. [13] analysed the effects of raindrop splash erosion and transversal width of soil erosion. Salles et al. [14] studied the effect of various simulated rain properties on soil detachment due to raindrop impact. Brodowski [15] analysed the influence of water layer depth on soil detachability. Part of the laboratory tests were carried out using a single drop instead of a rainfall simulator. Single drops were used for the studies of Al-Durrah and Bradford [16–18] and Nearing and Bradford [19]. Soil detachment by raindrops of varying kinetic energy was applied in Sharma et al. [8], while threshold energy, which is needed to detach particles from soil, was applied in Sharma and Gupta [20]. The effect of shear strength on soil splash was used by Mouzai and Bouhadef [21]. Drop impact pressure on soil splash saw application in Mouzai and Bouhadef [7], whereas the soil surface sealing effect was applied in Bradford et al. [22]. A model of soil detachment using single drop parameters was developed in Sharma et al. [23], as well as susceptibility to splash and mass of detached material [24].

To study the splash that occurred after the impact of a single water drop various measurement methods were used. In order to determine drop velocity Epema and Riezebos [25] used a time counter. A force transducer was used by Jayawardena and Rezaur [26] to measure the kinetic energy of rainfall or drop impact forces [27,28]. The laser diffraction method was used to measure aggregate size after splash by Legout et al. [29]. Optical microscope and image analysis of tracks of splashed particles recorded on blotting paper were used by Ryżak and Bieganowski, [30]. However, without doubt the largest group of splash studies are those conducted with the use of high-speed cameras. These measurement techniques facilitate observation of, inter alia, splash angle depending on soil strength [18], as well as the determination of the duration of the impact of a drop of water on the soil surface [31], rain splash of dry sand [32], and the effect of soil hydrophobicity [33] or artificial soils on the course of the splash phenomenon [34]. A set of three cameras was used to facilitate measurement of the movement of the ejected particles after water impact on a sand bed [11].

It should be stated that the name “single water drop” for the groups of methods (mentioned above) is not unequivocal. In fact, there are two groups of methods which are referred to by this name. Drops may fall singly, however in series (one after another) [7,30] or really singly—one drop hitting followed by an analysis of its impact [35,36]. The use of a really single drop, or single drops falling in a series, depends on the purpose of the experiment and its measurement capability (e.g., where the mass is to be measured, the amount of material transferred by one drop is unmeasurable). Awareness of how the drops are falling on to the surface is important because every drop modifies the properties of the soil in the place in which it fell. The moisture content and micro-relief of the surface (the geometry) are modified after the drop has fallen. Hence, in this type of measurement, is not possible to speak of repeatability but rather of the reproducibility of the measurements [37].

The aim of this study was to determine how the initial soil moisture content and soil moisture content modified by the following water drops influence the reproducibility of the splash phenomenon.

## Materials and Methods

Measurements were carried out on soil samples with different textures taken from the topsoil of two soil profiles in south eastern Poland [38]. After collection, these samples were dried at room temperature, sieved through a 2 mm sieve, and then humidified by mixing the dry soil with a respective amount of water. The water contents for both soils corresponded to the same pressure head: i) field water capacity ii) saturation and iii) the middle value. In this manner, the initial water content samples of investigated soils are presented in Table 1.

**Table 1:** Characteristics of soil material.

Soil		Particle size distribution(%, diameter mm)			Initial water content	
Type	Granulometric group	Sand2–0.05	Silt0.05–0.002	Clay <0.002	Pressure head [kPa]	(v %)
*Eutric Cambisol*	loamy silt	20.07	73.91	6.02	16	23
					3.1	28
					0.1	32
*Orthic Luvisol*	sandy loam	57.40	38.88	3.72	16	21
					3.1	26
					0.1	30

The moisturized soil was placed in the aluminium rings (with a diameter of 36 mm and a height of 10 mm) secured from the bottom by the chiffon. Then the samples were enclosed in a sealed container for 24 hours in order to equalize the moisture content of the sample before initiating the measurement of the splash phenomenon.

For each humidity setting, 13 rings were prepared with soil. One ring was used one time—i.e. after the falling of 10 drops it was not used again. Therefore, it can be stated that each ring was equal to one repetition of the experiment.

To make it easier for the reader to understand the text the following convention was adopted:—the term *drop* means the water which falls on the surface of the soil and causes the splash;—the term *droplet* means the water which is detached from the crown as the result of the splash.

Drops of water with a diameter of 4.2 mm (determined by the weight of drops and assuming the sphericity; SD = 0.002 mm) had been falling freely from a height of 1.5 m. Drops were created in the capillary, which was connected to a peristaltic pump dosing water at a rate of 9.6*10–7 m^3^/min. This allowed drops of constant frequency ca. 2s. The dispensing system was described in the work of Ryżak and Bieganowski [30]. The series of 10 drops (with a constant and uniform rate for all samples) was used for each sample (the ring with soil). It should be remembered that the local water content was changed after the falling of each water drop. The splash image was recorded after the fall of the 1^st^, 5^th^ and 10^th^ drop. The recorded images were analysed.

Measurements were recorded using two high-speed cameras (Vision Research MIRO M310) and recording data at 2000 frame per second. The samples were illuminated by two LED panels (back lighting) with dimensions of 0.6 x 0.6m, each of which guaranteed luminous flux of approximately 3500 lumens.

**Fig 1:**
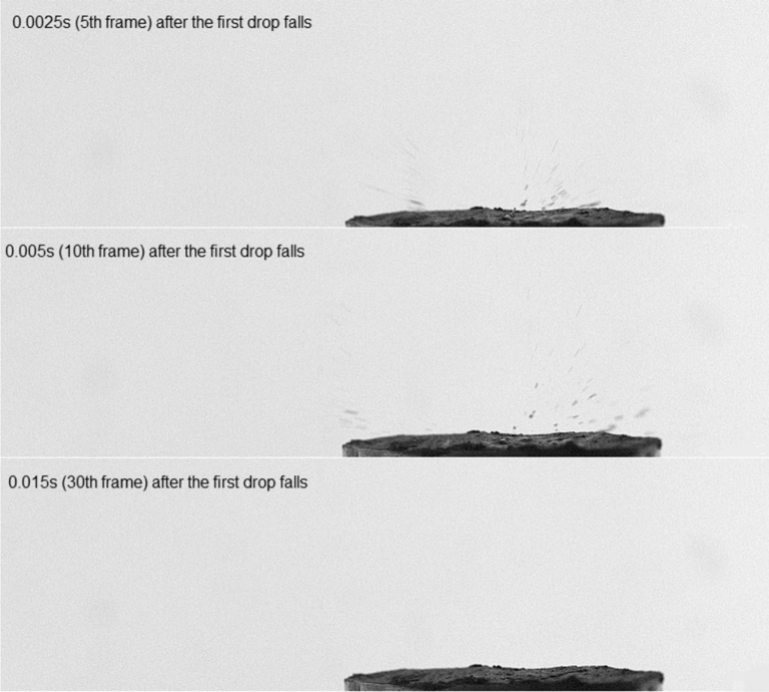
The dependence of the course of time (5^th^, 10^th^ and 30^th^ frames) of the splash phenomenon after the 1^st^ drop falls.

**Fig 2:**
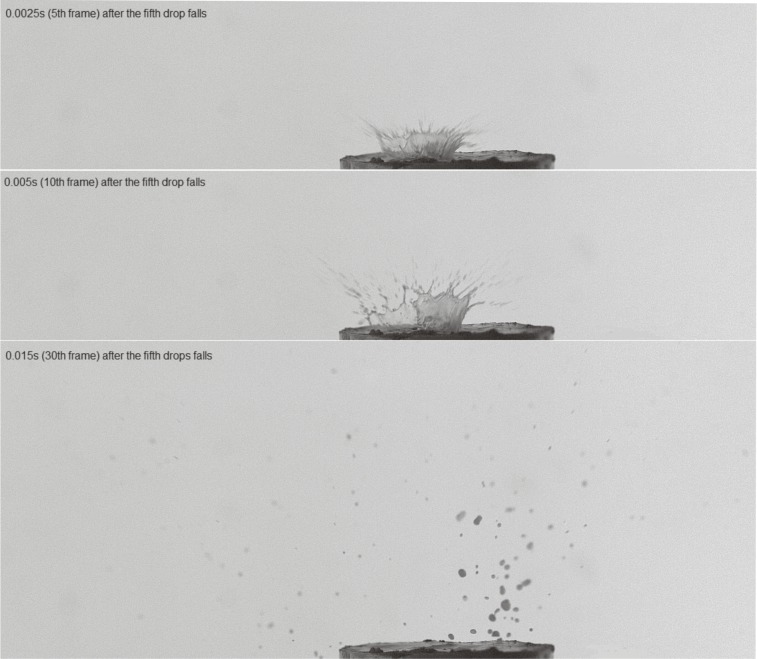
The dependence of the course of time (5^th^, 10^th^ and 30^th^ frames) of the splash phenomenon after the 5^th^ drop falls.

In the event of impact, the 1^st^ drop was analysed at a tenth of a frame (counting from the moment of contact of drops with the soil surface), corresponding to a time of 5*10^-3^s. In the case of impact for the 5^th^ and tenth drop, the 30^th^ frame was analysed (counting from the moment of contact of the drops with the soil surface), corresponding to a time of 15*10^-3^s. The differences in the time intervals arose from a different course of events between the two measurements. For the 1^st^ falling drop, there was no crown and the splash of individual particles phenomenon occurred so quickly that in 15*10^-3^s (30^th^ frame), there were no longer moving particles in the air. In the case of the 5^th^ and 10^th^ drop, a crown was formed. The reason for this was to collect water (suspension) at the point of incidence of consecutive drops—only water had not time to dwindle away into the sample. The dynamics of the phenomenon in the case of the formation of the crown shows that its breakdown and movement of particles after the collapse of the crown to be much smaller; hence, the need for extending the measurement time. In other words, if the crown was created during the splash, the analysis of the number and the surface of the particles was carried out after the crown rupture. To better illustrate the essence of the phenomenon, the different dynamics of splash have been summarized on Figs. 1 and 2 for one of the sample images of the repetitions.

The choice of measured parameters (number and surface of splashed soil particles and droplets and the width of the crown) was due to the possibility of the adopted measuring method. It was intended at the beginning of the experiment to measure the height of the craters created during the splash, however, this appeared impossible. The height of the crater was too small in relation to the resolution of the obtained images.

The number and the surface of the particles were analysed using the software Vision Assistant (National Instruments) through the following procedure: (i) calibrating photos by reading the size of the ring with the soil from the image and comparing the result with the actual magnitude of aluminium ring; (ii) digital processing of images using the options available in the program, such as Gaussian smoothing, convolution, conversion to binary image with background correction (threshold), incomplete closure of objects (proper close) and filling empty objects (filling holes); (iii) calculating the number and size of particles registered in the picture.

All recorded images were also analysed in terms of splash crown width (measured at the base), as formed after the fall of drops. Width from falling drops was measured at the 5^th^ frame, i.e., after a time of 2.5*10^-3^s following the fall of drops. This time was chosen to allow for crown "growth"; not that while there could be too large a crown, it was not allowed to rupture. The arbitrary choice of time does not matter, because the width of crown reproducibility was investigated using specific measurement conditions. Crown width was analysed manually, i.e., the width was determined by analysing the length of the segment that fit the base of the crown.

## Results and Discussion

### Number of splashed particles

**Fig 3:**
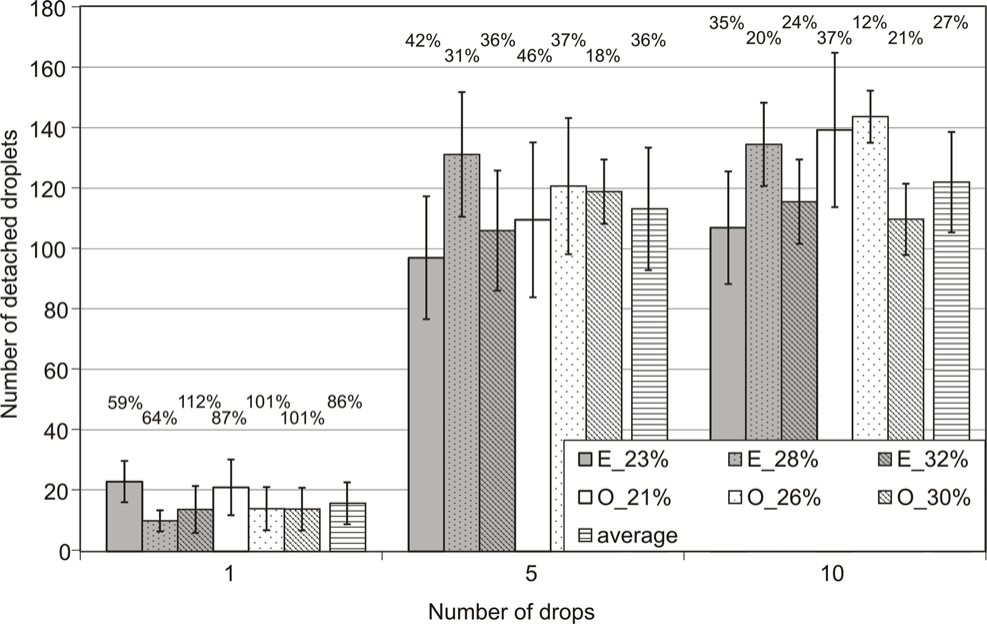
The dependence of the number of particles that have been splashed of the number of drops that have fallen on the sample at a given initial moisture content of the sample. Error bars represent sample standard deviation of 13 repetitions. The numbers of individual bars define the values of the coefficients of variation. O—represents *Ortic Luvisol*, E- represents *Eutric Cambisol*.

The registered number of splashed particles to the three different initial moisture content of soil samples tested is shown in Fig. 3 and S1 Table.

First of all, it should be noted on the basis of the image analysis that the term splashed particles include three types of particles: i) the soil particles detached from the soil surface, ii) the soil particles in the water droplets, and iii) the water droplets alone, detached from the water crown or reflected from the soil surface.

In the case of the fall of the 1^st^ drop, samples with the lowest initial moisture content were characterised by the largest number of splashed particles (above 20). For the remaining two higher initial soil humidity values, the numbers of splash particles were similar (average about 15). It should be noted that for *Eutric Cambisol* (a soil with finer fractions), the difference between the lowest initial humidity and the other two was greater than in the case of soil with a higher content of sand fraction.

After the fall of, respectively, the 5^th^ and 10^th^ drop, for both soils, the largest number of splashed particles occurred at the middle initial moisture content. For *Eutric Cambisol*, the number of particles was the smallest in the case of the lowest initial moisture content; for *Ortic Luvisol* this was the case at a high initial moisture content.

**Fig 4:**
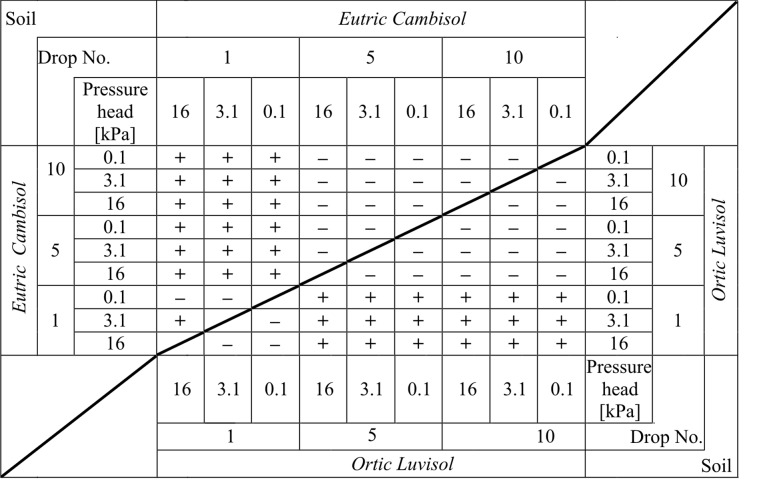
The statistical significance of differences between the mean numbers of splashed particles for the 1st, 5th and 10th drops for the different initial moisture levels for two soils. For *Eutric Cambisol* (left-upper part of the table) and separately for *Ortic Luvisol* (right-bottom part of the table). The significance was determined at the level α = 0.05. The symbol "+" indicates a statistically significant difference, while "-" indicates no statistically significant difference. Comparisons were made on a "peer-to-peer" basis. Note! A diagonal line separates two independent datasets that were not compared to one another.

**Fig 5:**
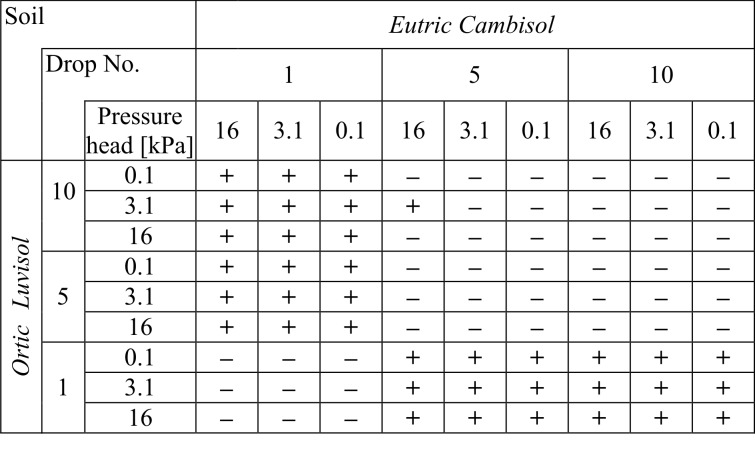
The statistical significance of differences between the mean numbers of splashed particles for the 1st, 5th and 10th drops for the different initial moisture levels for both investigated soil. The significance was determined at the level α = 0.05. The symbol "+" indicates a statistically significant difference, while "-" indicates no statistically significant difference. Comparisons were made on a "peer-to-peer" basis.

Full interpretation of the results of the splash presented in Fig. 3 was not possible without statistical analysis. As such, a given soil and the initial moisture content of each were analysed to find the statistical significance of differences between the two means (student t-test at significance level α = 0.05 peer method). A summary of the results of the tests are shown in Fig. 4. Furthermore, using the same test (and with the same level of significance), a comparison of the statistical significance of differences between the two test soils were made (Fig. 5).

By analysing data from both Figs. 4 and 5 it can be noted that significant differences occurred almost only when comparing the results of the first and the other drops. All other comparisons (except for two: *Eutric Cambisol*—pressure head 16 kPa, 5^th^ drop and *Ortic Luvisol*—pressure head 3.1 kPa, 10^th^ drop) showed no statistically significant difference for the mean number of particles. In other words, we can say that the phenomenon of splash occurs differently at the fall of the 1^st^ drop. The cause of this can be attributed to the fact that the 1^st^ drop fell on the surface of the soil, which had initial properties completely different than after the following drops. Of course, each next drop changed the properties of the soil. However, more drops resulted in less change of the properties. As the water content of the soil before the hit of the first water drop was different from the saturation, the fall of the 1^st^ drop caused the splash of individual particles of soil (see photos for the 1^st^ drop in Fig. 1). However, this water drop changed the water content in the place of the hitting and in the same time changed the geometry of the surface (the crater was created). This crater was not very visible in the pictures because these kinds of photos are not good tools to characterize this phenomenon; however, it should be clearly stated that it was. Therefore, the splash caused by the next drop took place in different conditions. The consequence was the different energy dissipation or disintegration [39].

The water content and structure of the soil surface changed with the fall of each subsequent drop until the next drop of water could no longer sink and formed a saturated layer and a micro-pool on the surface. The second fundamental change of the conditions was the appearance of the water layer on the surface of the soil. It was difficult to indicate precisely which water drop caused the saturation of the soil in both investigated soils (it was impossible to monitor this by photos taken from the side). However, the creating of the water crown after the 5^th^ drop was the evidence that saturation had to have taken place before this drop (Fig. 2). Even a very thin layer of water on the soil surface completely changes the conditions of the splash—it can enhance the force of impact of drops and increase the amount of splashed material in comparison to the soil without a water layer [40].

The change of the conditions in the place of the impact of the drop did not change dramatically after the appearance of a micro-pool. Therefore, qualitatively, the impact of the 5^th^ and 10^th^ drops did not differ much, which was the reason for the lack of statistically significant differences.

Complementing the analysis of the results shown in Fig. 3, it is worth referring to the scatter of the results. Spread may be determined by the so-called coefficient of variation, or CV (the ratio of the standard deviation to the mean expressed in percent). The highest value of the coefficients of variation (i.e., the smallest repeatability) was the 1^st^ drop, with CV values between 59% and 101%, with an average for all soils of 86%. The lowest values of the coefficient of variation were recorded for the 10^th^ drop, with CV values between 12% and 37% with an average value for all soils of 27%. In general, one can say that with every drop, the splash phenomenon occurred more consistently.

### Surface of splashed particles

**Fig 6:**
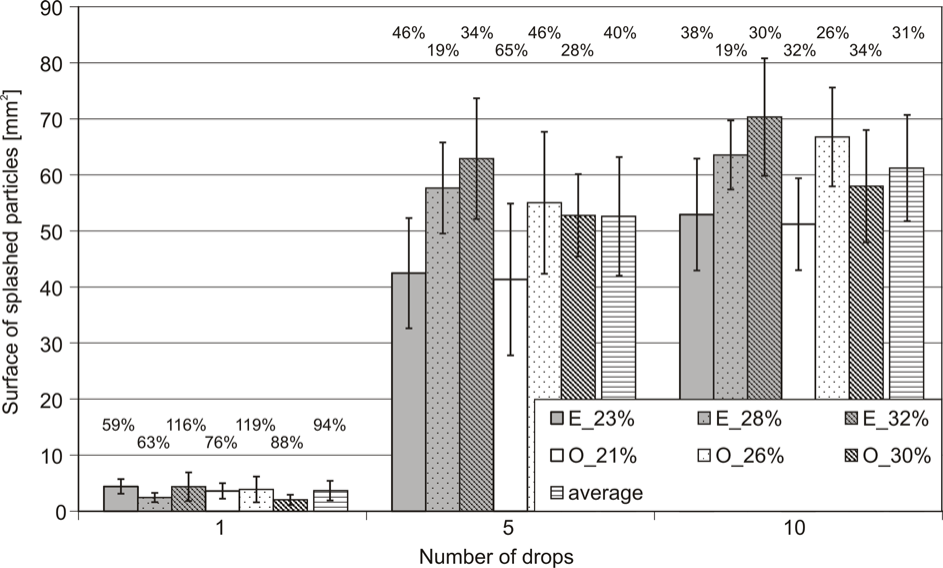
The dependence of the surface of splashed particles to the number of drops that dropped on the sample at a given initial moisture content of the sample. Error bars represent sample standard deviation of 13 replicates. The numbers of individual bars define the values of the coefficients of variation. O—represents *Ortic Luvisol*, E- represents *Eutric Cambisol*.

The summary surface area of the splashed particles (which is the sum of surface areas of individual particles) can be treated as the measure of the splash phenomenon. The registered surfaces of detached particles to the three different pressure head of soil samples investigated are shown in Fig. 6 and S2 Table. In order to briefly describe the results obtained for surface analysis of particles, it should be noted that, in practice, the above situation is repeated, i.e., all the trends described for the analysis of the number of splashed particles are applicable for the analysis of the surface. Similarly, statistically significant differences almost only occur when comparing the splash caused by the 1^st^ drop, but there is no such difference between the 5^th^ and 10^th^ drops when considering different soils. Again, the greatest dispersion occurred in the case of the 1^st^ drop and the lowest at the 10^th^ drop. Thus, all the conclusions presented in the previous sub-section also apply in relation to the analysis of the surface of the detached drops.

**Fig 7:**
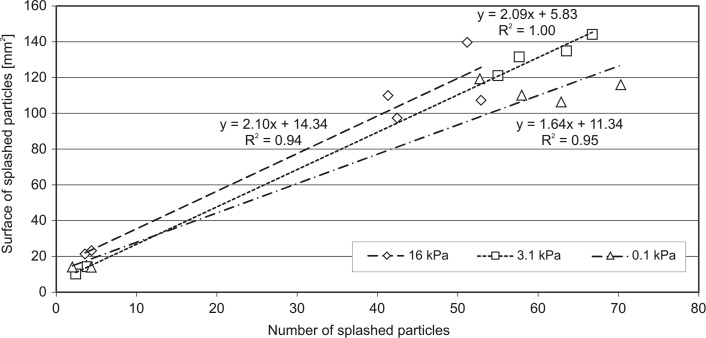
Correlation between the number of splashed particles and their surface for different pressure heads of both investigated soils.

The analysis of Figs. 3 and 6 shows, indirectly, the correlation between the number of splashed particles and their surface area is significant. Fig. 7 and S3 Table shows this clearly.

It can be seen from Fig. 7 that the correlation between the number of splashed particles and their surface area is practically independent from the initial pressure head (the slopes and determination coefficients are at a similar level). Therefore, all points presented in Fig. 7 were treated as one population and the straight line was interpolated to all these data. The following equation of this line was obtained: y = 1.87x + 12.79 and R² was equal to 0.93.

### Crown width

**Fig 8:**
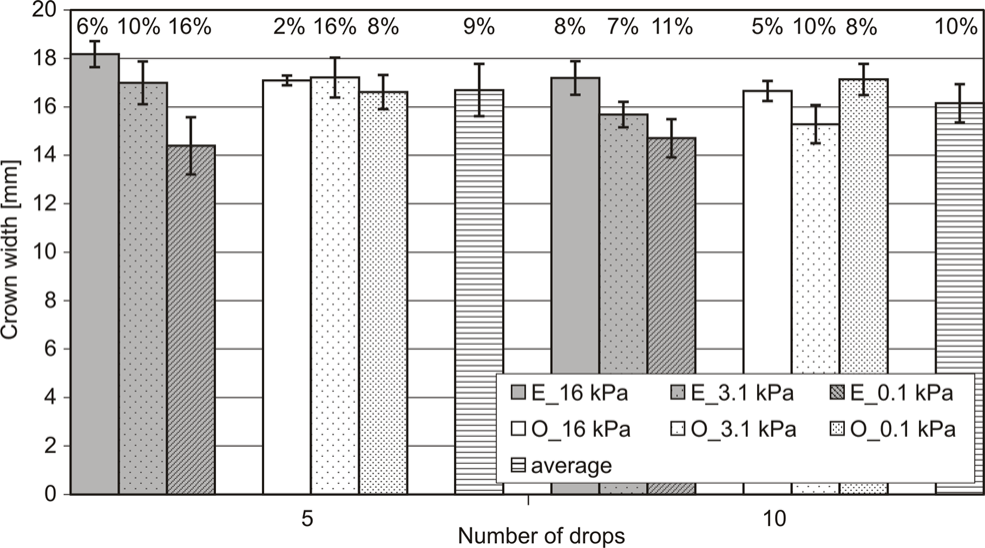
Crown widths specified in 2.5*10^-3^s (5 frame) after the collapse of the 5^th^ and 10^th^ drops. Error bars represent sample standard deviation of 13 replicates. The number of individual bars defines the values of the coefficients of variation. O—represents *Ortic Luvisol*, E- represents *Eutric Cambisol*.

Selection of the width of the crown, which came after the fall of the 5^th^ and 10^th^ drops, is shown in Fig. 8. There are no results for the 1^st^ drop, due to the absence of the crown following the fall of the 1^st^ drop. Therefore, it is difficult to describe the influence of the initial pressure head on the crown width. The crown was created when the micro-pool was created at the surface of the soil—in other words the soil in the vicinity of the place where the water drops was saturated.

**Fig 9:**
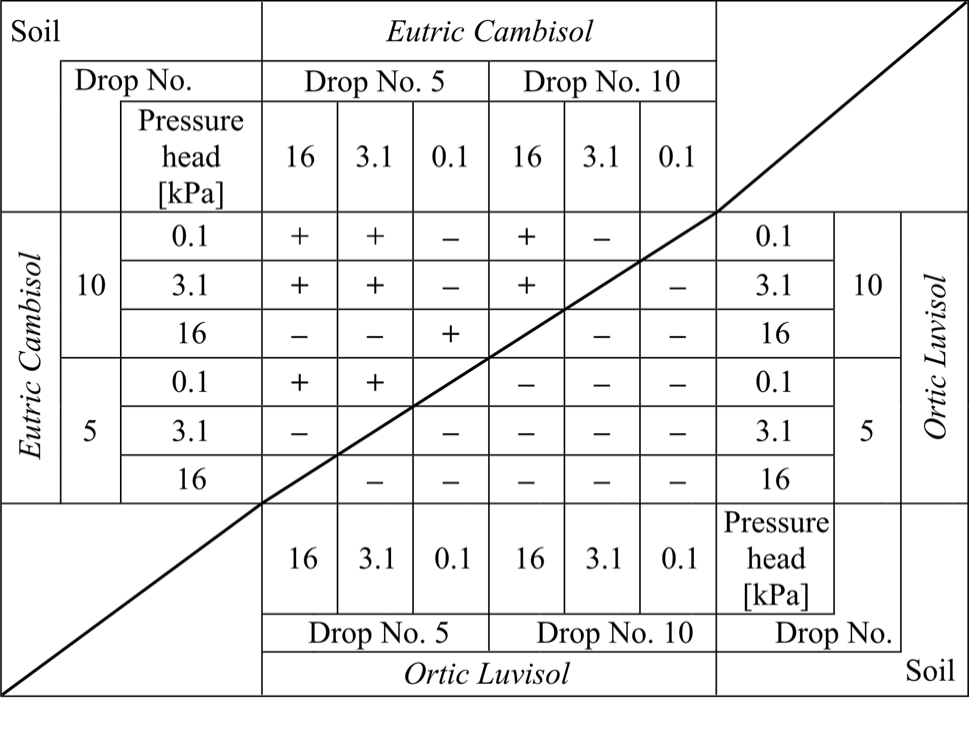
Determination of the statistical significance of differences (α significance level = 0.05) for the values shown in Fig. 6 formed between the crown widths of the 5^th^ and after the impact of the 10^th^ drops at different initial moisture contents for both soils. The symbol "+" indicates a statistically significant difference, while "-" indicates no statistically significant difference. Comparisons were made on a "peer-to-peer" basis.

By analysing the data from the chart in Fig. 8 and S4 Table it can be seen that the repeatability of the phenomenon is much larger than in the case of the number and surface of splashed particles. Repeatability is expressed both by the similar values in crowns width (between 14 and 18 mm, with an average of slightly more than 16 mm) and by much smaller coefficients of variation (CV to within 2% to 16%, with an average of about 10% to both soils). It should be noted, however, that the width of the crown differed between the two soils. The greater spread of results was seen for *Eutric Cambisol* (contains more silt fraction). Confirmation of this observation can be found in Fig. 9, where more than half of the soil variations were statistically significant.

In addition, for *Eutric Cambisol*, we observed a trend that the larger the initial humidity of the sample the smaller the crown was, but the scatter of the results increased (Fig. 8). Such a trend was not observed for *Orthic Luvisol*. For *Orthic Luvisol*, a statistically significant difference was observed in the comparison shown in Fig. 9. This difference between the two soils may be explained on the basis of differences in particle size distribution. *Eutric Cambisol* contained far finer fraction than *Ortic Luvisol* (Table 1). Hence, the speed of the subsequent infiltration of water drops was higher at *Ortic Luvisol* (sandy loam). This in turn provided more consistent surface conditions for the soil at the time of incidence of the subsequent drops. However, *Eutric Cambisol* (loamy silt), sinking was slower; therefore, more water gathered on the soil surface. This made a micro-pool form on the surface of *Eutric Cambisol*, i.e. in this case, a greater film of water covered the soil surface. It can therefore be concluded that the higher the water film on the surface, the less the width of the forming crown is. This was confirmed by the results of a separate measurement carried out for the system: a drop of water hit the surface of a water (water column of 1 cm) situated in the vessel; the height of the falling drop was the same as in the experiment with soil, i.e., 1.5 m. In this case, there was a crown width of 10.3 mm, which was less than the minimum width of the crown for *Eutric Cambisol*.

## Conclusions

The main conclusion from the described experiments is the statement that the splash phenomenon occurs on the soil surface which is unsaturated is significantly different than that which forms on the surface of saturated soil. It can be said that the splash phenomenon depends on the initial soil pressure head in this context.

The confirmation of this statement can be found in the observation that results of the splash caused by the 1^st^ drop striking the surface differed from the results of the splash caused by the impact of subsequent drops. This difference was due to the fact that less moisture was present at the time of incidence of the 1^st^ drop. With each subsequent drop the local water content increased, up to saturation.

After reaching saturation of the soil surface, a micro-pool formed, while during the splash a crown formed. Under the conditions of the experiment the following was shown: for soil—*Eutric Cambisol* (loamy silt) and *Orthic Luvisol* (sandy loam)—the lowest humidity test output (equivalent to pressure head 16 kPa) crown always formed after the 5^th^ drop.

With each drop impinging on the same place, the splash phenomenon became more reproducible, i.e., the number of particles of soil and/or water that splashed became more similar to one another. The numbers and the surface structure of the splashed particles were similar after the impact of the 5^th^ and 10^th^ water drops. It can be said in this context, that when the time interval between the following drops is short enough, a few water drops, which hit the same place, are able to modify the water content of the soil to such an extent (making it saturated) that the influence of initial moisture content disappears. However, it can be expected that when the time interval increases and/or the water permeability of the soil is higher, the number of the water drops which would saturate the impact site would increase.

The number of detached particles during the splash was strongly correlated with its surface area; therefore, the summary surface area of the splashed particles (which is the sum of surface areas of individual particles) can be treated as the measure of the efficiency of the splash phenomenon.

As the scatter of the results (expressed for instance as the number of splashed particles) of the splash phenomenon is so high that it is impossible to find differences between soils, the width of the crown was soil dependent. The reproducibility of the width of the crown resulting in the splash in the case of loamy silt soil was much less than that of the crown resulting in the case of a sandy loam soil. However the higher the water film on the surface, the less the width of the forming crown was (the widest crown obtained in the conditions of the experiment was approximately 18 mm). Formation of the smallest crown width was observed in the case of the splash drop on the water surface (about 10 mm).

## Supporting Information

S1 Table The number of particles that have been splashed by drops that have fallen on the sample at a given initial moisture content of the sample. O—represents *Ortic Luvisol*, E- represents *Eutric Cambisol*, SD—represents sample standard deviation of 13 repetitions.
(DOC)

S2 Table The surface of particles that have been splashed by drops that have fallen on the sample at a given initial moisture content of the sample. O—represents *Ortic Luvisol*, E- represents *Eutric Cambisol*, SD—represents sample standard deviation of 13 repetitions.
(DOC)

S3 Table The surface and number of splashed particles for different pressure heads of both investigated soils. (DOC)

S4 Table The width of crown specified in 2.5*10^-3^s (5 frame) after the collapse of the 5^th^ and 10^th^ drops. O—represents *Ortic Luvisol*, E- represents *Eutric Cambisol*, SD—represents sample standard deviation of 13 repetitions.
(DOC)
