# Supplementary material for: Effect of Soil Moisture Content on the Splash Phenomenon Reproducibility
Source: PLoS One. 2015 Mar 18;10(3):e0119269. doi: 10.1371/journal.pone.0119269 (PMC4364956; doi:10.1371/journal.pone.0119269)
Supplement: S1 Table — O—represents Ortic Luvisol, E- represents Eutric Cambisol, SD—represents sample standard deviation of 13 repetitions. (DOC) [file pone.0119269.s001.doc]

SUPPORTING TABLE S1 for

**Effect of soil moisture content on the splash phenomenon reproducibility**

Magdalena Ryżak, Andrzej Bieganowski, Cezary Polakowski

**S1 Table.** **The number of particles that have been splashed by drops that have fallen on the sample at a given initial moisture content of the sample.** O – represents *Ortic Luvisol,* E-represents *Eutric Cambisol*, SD – represents sample standard deviation of 13 repetitions.

| Number of drops | Sample name | Number of particles | ½*SD |
| --- | --- | --- | --- |
| 1 | E_16kPa | 23.11 | 6.84 |
|  | E_3.1 kPa | 10.11 | 3.52 |
|  | E_0.1 kPa | 13.91 | 7.76 |
|  |  |  |  |
|  | O_16 kPa | 21.20 | 9.23 |
|  | O_3.1 kPa | 14.17 | 7.12 |
|  | O_0.1 kPa | 14.00 | 7.09 |
|  |  |  |  |
|  | average | 15.93 | 6.93 |
|  |  |  |  |
| 5 | E_16kPa | 97.25 | 20.34 |
|  | E_3.1 kPa | 131.50 | 20.66 |
|  | E_0.1 kPa | 106.27 | 19.89 |
|  |  |  |  |
|  | O_16 kPa | 109.83 | 25.67 |
|  | O_3.1 kPa | 121.00 | 22.57 |
|  | O_0.1 kPa | 119.17 | 10.61 |
|  |  |  |  |
|  | average | 113.47 | 20.28 |
|  |  |  |  |
| 10 | E_16kPa | 107.23 | 18.65 |
|  | E_3.1 kPa | 134.82 | 13.78 |
|  | E_0.1 kPa | 115.85 | 13.95 |
|  |  |  |  |
|  | O_16 kPa | 139.60 | 25.55 |
|  | O_3.1 kPa | 144.00 | 8.62 |
|  | O_0.1 kPa | 110.00 | 11.79 |
|  |  |  |  |
|  | average | 122.31 | 16.65 |
