# Supplementary material for: Effect of Soil Moisture Content on the Splash Phenomenon Reproducibility
Source: PLoS One. 2015 Mar 18;10(3):e0119269. doi: 10.1371/journal.pone.0119269 (PMC4364956; doi:10.1371/journal.pone.0119269)
Supplement: S2 Table — O—represents Ortic Luvisol, E- represents Eutric Cambisol, SD—represents sample standard deviation of 13 repetitions. (DOC) [file pone.0119269.s002.doc]

SUPPORTING TABLE S2 for

**Effect of soil moisture content on the splash phenomenon reproducibility**

Magdalena Ryżak, Andrzej Bieganowski, Cezary Polakowski

**S2 Table. The surface of particles that have been splashed by drops that have fallen on the sample at a given initial moisture content of the sample.** O – represents *Ortic Luvisol,* E-represents *Eutric Cambisol*, SD – represents sample standard deviation of 13 repetitions.

| Number of drops | Sample name | Surface of particles [mm^2^] | ½*SD |
| --- | --- | --- | --- |
| 1 | E_16kPa | 4.43 | 1.31 |
|  | E_3.1 kPa | 2.45 | 0.83 |
|  | E_0.1 kPa | 4.39 | 2.55 |
|  |  |  |  |
|  | O_16 kPa | 3.61 | 1.38 |
|  | O_3.1 kPa | 3.88 | 2.30 |
|  | O_0.1 kPa | 2.03 | 0.90 |
|  |  |  |  |
|  | average | 3.66 | 1.75 |
|  |  |  |  |
| 5 | E_16kPa | 42.47 | 9.85 |
|  | E_3.1 kPa | 57.65 | 8.12 |
|  | E_0.1 kPa | 62.90 | 10.78 |
|  |  |  |  |
|  | O_16 kPa | 41.34 | 13.53 |
|  | O_3.1 kPa | 55.02 | 12.65 |
|  | O_0.1 kPa | 52.77 | 7.36 |
|  |  |  |  |
|  | average | 52.61 | 10.57 |
|  |  |  |  |
| 10 | E_16kPa | 52.92 | 9.99 |
|  | E_3.1 kPa | 63.55 | 6.16 |
|  | E_0.1 kPa | 70.31 | 10.49 |
|  |  |  |  |
|  | O_16 kPa | 51.19 | 8.20 |
|  | O_3.1 kPa | 66.75 | 8.81 |
|  | O_0.1 kPa | 57.97 | 10.02 |
|  |  |  |  |
|  | average | 61.21 | 9.47 |
