# Supplementary material for: Effect of Soil Moisture Content on the Splash Phenomenon Reproducibility
Source: PLoS One. 2015 Mar 18;10(3):e0119269. doi: 10.1371/journal.pone.0119269 (PMC4364956; doi:10.1371/journal.pone.0119269)
Supplement: S3 Table — (DOC) [file pone.0119269.s003.doc]

SUPPORTING TABLE S3 for

**Effect of soil moisture content on the splash phenomenon reproducibility**

Magdalena Ryżak, Andrzej Bieganowski, Cezary Polakowski

**S3 Table. The surface and number of splashed particles for different pressure heads of both investigated soils.**

|  | Surface [mm^2^] | Number |
| --- | --- | --- |
| pressure head 16 kPa | 4.43 | 23.11 |
|  | 42.47 | 97.25 |
|  | 52.92 | 107.23 |
|  | 3.61 | 21.20 |
|  | 41.34 | 109.83 |
|  | 51.19 | 139.60 |
|  |  |  |
| pressure head 3.1 kPa | 2.45 | 10.11 |
|  | 57.65 | 131.50 |
|  | 63.55 | 134.82 |
|  | 3.88 | 14.17 |
|  | 55.02 | 121.00 |
|  | 66.75 | 144.00 |
|  |  |  |
| pressure head 0.1 kPa | 4.39 | 13.91 |
|  | 62.90 | 106.27 |
|  | 70.31 | 115.85 |
|  | 2.03 | 14.00 |
|  | 52.77 | 119.17 |
|  | 57.97 | 110.00 |
