# Supplementary material for: Effect of Soil Moisture Content on the Splash Phenomenon Reproducibility
Source: PLoS One. 2015 Mar 18;10(3):e0119269. doi: 10.1371/journal.pone.0119269 (PMC4364956; doi:10.1371/journal.pone.0119269)
Supplement: S4 Table — O—represents Ortic Luvisol, E- represents Eutric Cambisol, SD—represents sample standard deviation of 13 repetitions. (DOC) [file pone.0119269.s004.doc]

SUPPORTING TABLE S4 for

**Effect of soil moisture content on the splash phenomenon reproducibility**

Magdalena Ryżak, Andrzej Bieganowski, Cezary Polakowski

**S4 Table.** **The width of crown specified in 2.5*10^-3^s (5 frame) after the collapse of the 5^th^ and 10^th^ drops.** O – represents *Ortic Luvisol,* E-represents *Eutric Cambisol*, SD – represents sample standard deviation of 13 repetitions.

| Number of drops | Sample name | Crown width [mm] | ½*SD |
| --- | --- | --- | --- |
| 5 | E_16kPa | 18.18 | 0.54 |
|  | E_3.1 kPa | 16.99 | 0.89 |
|  | E_0.1 kPa | 14.39 | 1.18 |
|  |  |  |  |
|  | O_16 kPa | 17.09 | 0.21 |
|  | O_3.1 kPa | 17.21 | 0.82 |
|  | O_0.1 kPa | 16.61 | 0.71 |
|  |  |  |  |
|  | average | 16.69 | 1.08 |
|  |  |  |  |
| 10 | E_16kPa | 17.19 | 0.69 |
|  | E_3.1 kPa | 15.68 | 0.53 |
|  | E_0.1 kPa | 14.70 | 0.79 |
|  |  |  |  |
|  | O_16 kPa | 16.65 | 0.42 |
|  | O_3.1 kPa | 15.28 | 0.79 |
|  | O_0.1 kPa | 17.13 | 0.65 |
|  |  |  |  |
|  | average | 16.15 | 0.79 |
